# Metabolic Profile of Patients with Long COVID: A Cross-Sectional Study

**DOI:** 10.3390/nu15051197

**Published:** 2023-02-27

**Authors:** Daniel Carvalho de Menezes, Patrícia Danielle Lima de Lima, Igor Costa de Lima, Juliana Hiromi Emin Uesugi, Pedro Fernando da Costa Vasconcelos, Juarez Antônio Simões Quaresma, Luiz Fábio Magno Falcão

**Affiliations:** 1 Center for Biological Health Sciences, State University of Pará (UEPA), Belém 66087-670, Brazil; 2 School of Medicine, São Paulo University (USP), São Paulo 01246903, Brazil; 3 Tropical Medicine Center, Federal University of Pará (UFPA), Belém 66055-240, Brazil

**Keywords:** long COVID, cardiometabolic risk factors, laboratory markers

## Abstract

A significant proportion of patients experience a wide range of symptoms following acute coronavirus disease 2019 (COVID-19). Laboratory analyses of long COVID have demonstrated imbalances in metabolic parameters, suggesting that it is one of the many outcomes induced by long COVID. Therefore, this study aimed to illustrate the clinical and laboratory markers related to the course of the disease in patients with long COVID. Participants were selected using a clinical care programme for long COVID in the Amazon region. Clinical and sociodemographic data and glycaemic, lipid, and inflammatory screening markers were collected, and cross-sectionally analysed between the long COVID-19 outcome groups. Of the 215 participants, most were female and not elderly, and 78 were hospitalised during the acute COVID-19 phase. The main long COVID symptoms reported were fatigue, dyspnoea, and muscle weakness. Our main findings show that abnormal metabolic profiles (such as high body mass index measurement and high triglyceride, glycated haemoglobin A1c, and ferritin levels) are more prevalent in worse long COVID presentations (such as previous hospitalisation and more long-term symptoms). This prevalence may suggest a propensity for patients with long COVID to present abnormalities in the markers involved in cardiometabolic health.

## 1. Introduction

There have been millions of confirmed cases of coronavirus disease 2019 (COVID-19) globally. Based on recent reviews, severe acute respiratory syndrome coronavirus 2 (SARS-CoV-2) infection may be related to not only the involvement of respiratory organs but also non-respiratory systems, causing problems such as male infertility [1], liver disease [2] and gastrointestinal disease [3], indicating a multiorgan involvement by COVID-19.

A significant proportion of patients continue to experience a wide range of physical, mental, and psychological symptoms following their acute illness, characterising a post-acute involvement commonly referred to as long COVID [4,5]. Patients with this long-term condition may have persistent symptoms for more than four weeks after the acute phase of COVID-19 [6,7,8,9,10].

Metabolic dysfunction related to metabolic syndrome (MS) is suggested to be one of the many outcomes induced by long COVID [11,12]. Laboratory analyses of long COVID have demonstrated imbalances in cardiometabolic parameters, such as lipid, glycaemic, and obesity-related markers [7]. In addition, monitoring inflammatory biomarkers such as the erythrocyte sedimentation rate (ESR), serum ferritin, and C-reactive protein (CRP), as well as other markers associated with metabolism, can help to understand the evolution and maintenance of long COVID symptoms [13].

Thus, this study aimed to determine the clinical and laboratory profiles of patients with long COVID and explore the interactions between metabolic abnormalities and long-term outcomes. Our main findings show that abnormal metabolic profiles are more prevalent in worse long COVID presentations.

## 2. Materials and Methods

This is a prospective, cross-sectional, observational study that was conducted in strict accordance with the principles of the Declaration of Helsinki and reported according to the Strengthening the Reporting of Observational Studies in Epidemiology (STROBE) guidelines for observational studies. This study was approved by the Ethics Committee for Research Involving Human Beings of the State University of Pará (Protocol No. 4.252.664). All the participants provided written informed consent.

A total of 258 adults (≥18 years old) of both sexes diagnosed with long COVID were selected between March 2020 and December 2021, following the order of voluntary registration in a clinical follow-up programme for patients with long COVID in the Brazilian Amazon region. The following criteria were considered for the diagnosis of long COVID: (I) the acute symptomatic phase of COVID-19 confirmed by a reverse transcription–quantitative polymerase chain reaction (RT-qPCR), with symptoms consistent with COVID-19 not attributable to any other cause; and (II) at least one long-term symptom related to COVID-19, such as cough, dyspnoea, chest pain, muscle weakness, loss of balance, tremor, fatigue, muscle pain, headache, visual disturbances, insomnia, and/or lower limb oedema, not attributable to another differential diagnosis, for at least four weeks after the onset of symptoms. The time interval between symptom onset and diagnostic confirmation ranged up to 3 days, while the interval between the diagnosis of COVID-19 and the long COVID patients’ clinical and laboratory evaluation ranged from 32 to 632 days.

**Figure 1:**
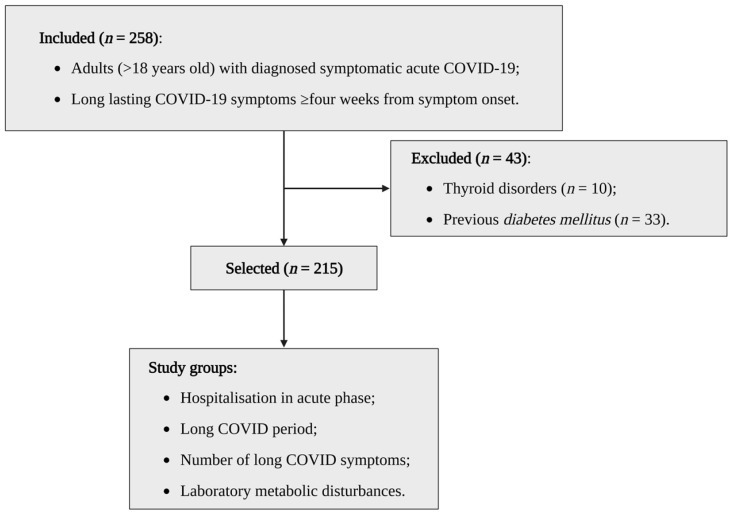
Flowchart of patient recruitment and study group allocation. Created with BioRender.com (accessed on 23 February 2023).

We excluded 10 patients with thyroid disorders (hyperthyroidism or hypothyroidism) and 33 patients with diabetes since these are conditions that could interfere with the values of metabolic laboratory markers. The use of drugs that could alter these levels, such as corticosteroids, was also considered as an exclusion criterion, but none of the patients included used medications at the time of data collection. The other 215 patients were allocated into the following groups: (i) according to the outcomes associated with COVID-19: “hospitalisation in acute phase”; “long COVID period” (period from the onset of symptoms to the time of data collection); and “number of long COVID symptoms”; (ii) according to laboratory abnormalities related with the metabolic profile: “laboratory metabolic disturbances” (fasting blood glucose (FBG): ≥126 mg/dL and/or glycated haemoglobin A1c (HbA1c) ≥6.5% and/or low-density cholesterol (LDL-C) ≥160 mg/dL and/or triglycerides ≥200 mg/ dL) (Figure 1). These groups were composed of different patients, and data collection was carried out in a single step.

After fasting for at least 8 h, 3 mL of blood was collected in two Vacuette^®^ tubes (Greiner Bio-One, Kremsmünster, Austria), one with ethylenediaminetetraacetic acid anticoagulant, for the analysis of whole blood, which consisted of the quantification of HbA1c and ESR; and another tube with a separator gel and clot activator for the analysis of serum as follows: LDL-C, high-density cholesterol (HDL-C), total cholesterol, triglycerides, FBG, serum ferritin, and CRP. As a reference parameter, the values used by the respective reagent manufacturers were adopted and classified as “desirable” and “risk” in relation to the metabolic profile (Supplementary Table S1). Of the 215 patients, 65 were not tested for HbA1c.

Soon after, with a maximum interval of 24 h, the patients were interviewed to obtain their demographic and clinical data, such as name, age, sex, monthly income, job status, smoking status, long COVID symptoms and period, comorbidities, hospital admission during the acute phase, length of stay, and the medications used. In addition, systolic blood pressure (SBP) and diastolic blood pressure (DBP) were measured at rest, and the patient’s weight and height were measured, followed by the calculation of the body mass index (BMI). Of the 215 patients, 49 did not report their job status and monthly income.

The clot activator tube was centrifuged for 5 min at 3000 rpm (Daiki 80-2B centrifuge, Ionlab, Araucária, Brazil), followed by the determination of the quantitative values of serum metabolic markers using a CMD 600 semi-automatic compact auto-analyser with a reagent line for clinical chemistry (Wiener Lab Group, Rosario, Argentina). Lipid markers were quantified in addition to glucose and serum ferritin. Whole-blood samples were analysed using the same equipment, and HbA1c levels were quantified. The ESR was also determined in whole-blood samples after 1 h of rest. The presence of CRP (Latex PCR SD LabTest, Lagoa Santa, Brazil) was qualitatively analysed.

The collected data were tabulated in an Excel™ spreadsheet (Microsoft Corporation, Redmond, DC, USA) and analysed using GraphPad Prism™ software version 8.4.3 (GraphPad Software, San Diego, CA, USA). Data normality was assessed with the D’Agostino Pearson test, using the mean and standard deviation for data description. For comparisons between groups, the Mann–Whitney and chi-square tests were used to compare the variables without a normal distribution, while an analysis of variance (ANOVA) was used for comparisons with a normal distribution. Multiple logistic regression analysis was used to verify the predictors and associations between different study variables. Linear correlation between metabolic and inflammatory markers was evaluated using Pearson’s correlation coefficient in the long COVID outcome groups. A two-tailed *p*-value of <0.05 was considered statistically significant.

## 3. Results

Of the 215 patients included, 138 were female, and 161 were aged between 18 and 59 years, with 78 hospitalisations during the acute phase of COVID-19. The main symptoms reported were fatigue (*n* = 184), dyspnoea (*n* = 178), and muscle weakness (*n* = 168). The long COVID period ranged from 32 to 632 days, with an average of 247.7 days (standard deviation (SD) 151.2). The most common self-reported comorbidity was hypertension (*n* = 64), and 89 patients had a BMI suggestive of obesity (≥30 kg/m^2^). Even though most laboratory mean values remained close to the desirable reference thresholds, the ESR levels were increased. Patients with laboratory metabolic disturbances (*n* = 102) reported more muscle weakness (*n* = 88) and higher mean ferritin levels and age (Table 1).

Of the 166 participants who reported their monthly income and job status, 56.6% (*n* = 94) performed paid activities, with 65.9% (*n* = 62) being employees, business owners, or military personnel, and 34% (*n* = 32) being self-employed. Among the remaining 43.3% (*n* = 72) who did not work, 62.5% (*n* = 45) were unemployed, 13.8% (*n* = 10) were retired, 16.6% (*n* = 12) were homemakers, and 6.9% (*n* = 5) were students. Regarding those who had a monthly income (employees, business owners, military personnel, self-employed, and retirees, *n* = 104), 12.5% (*n* = 13) had an income of up to USD 200, 36.5% (*n* = 38) had an income between USD 200 and 400, 35.5% (*n* = 37) had an income between USD 400 and 1000, 12.5% (*n* = 13) had an income between USD 1000 and 2000, and 2.8% (*n* = 3) had an income of more than USD 2000.

The mean triglyceride and ferritin levels were higher in hospitalised patients (*n* = 78) than in non-hospitalised patients (*n* = 137). The group of patients with up to 90 days of long COVID (*n* = 35) showed abnormalities in HbA1c and ferritin levels when compared to its counter-group (>90 days, *n* = 180). On the other hand, patients with more than 365 days of long COVID (*n* = 45) had higher mean BMI than those with a period of long COVID of less than a year (*n* = 170). Furthermore, the mean measurements of BMI and HbA1c mean values were higher in the group of patients with more than six simultaneous symptoms (*n* = 121) when compared to its counter-group (up to six simultaneous symptoms, *n* = 94). In contrast, ferritin levels were higher in patients with up to six symptoms (*n* = 94), compared with the group with more than six symptoms (*n* = 121) (Table 2). The group of patients with up to 90 days of long COVID (*n* = 35) presented a mean of 7 (SD 2.8) simultaneous symptoms, while patients with more than 365 days of long COVID (*n* = 45) showed a mean of 6.8 (SD 2.6) symptoms.

**Figure 2:**
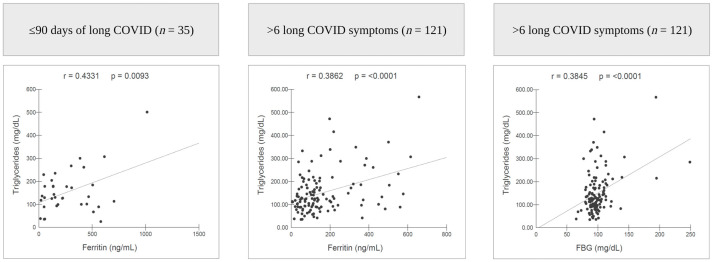
Pearson’s correlation coefficient (r). FBG, fasting blood glucose. Created with BioRender.com (accessed on 23 February 2023).

Hospitalisation and higher HbA1c and ferritin levels were associated with a long COVID period of up to 90 days. High BMI was associated with long COVID for more than 365 days. In addition, female sex, hospitalisation, and high BMI increased the risk of presenting more than six simultaneous symptoms in patients with long COVID. Fatigue was associated with a long COVID period of over 90 days (Table 3). A stronger correlation between triglycerides and FBG levels was observed, as was the case for triglycerides and ferritin in patients with more than six symptoms. Regarding the long COVID period, the correlation between triglycerides and ferritin was stronger in patients up to 90 days (Figure 2).

## 4. Discussion

Most of the 215 patients included were females aged 18–59. Seventy-eight of the patients had been hospitalised during the COVID-19 acute phase, and the main long COVID symptoms reported were fatigue, dyspnoea, and muscle weakness. Among the hospitalised patients, the mean levels of triglycerides and ferritin were higher than those in the non-hospitalised group. In patients with a shorter period of long COVID, most quantitative laboratory tests, especially HbA1c and ferritin levels, were higher. Furthermore, BMI and HbA1c levels were higher in patients with more than six symptoms. Female sex, hospitalisation, and a high BMI were associated with more symptoms. Finally, triglyceride levels were correlated with FBG and ferritin levels in the worst long COVID outcomes.

The most commonly reported long COVID symptoms in recent studies are fatigue, dyspnoea, chest pain, and muscle weakness, among other non-specific manifestations [4,11]. This study demonstrated a similar profile of involvement, with fatigue being the most frequent symptom. In addition, most of the affected population consisted of women aged approximately 50 years, in agreement with several recent investigations [10,14,15,16].

Patients who had been hospitalised during the COVID-19 acute phase showed abnormal levels of triglycerides and ferritin. Although there is evidence that hospitalisation during the onset of symptoms is a risk factor for long COVID susceptibility and severity [17,18], it is unclear whether hospitalisation is directly responsible for the abnormality in the markers mentioned above and how this interaction occurs considering the vast multiorgan pathophysiology of long COVID. However, the non-specific harmful effects of hospitalisation certainly influence the long-term abnormalities in these markers, as demonstrated in the current study.

Our results suggest that patients with a shorter period of COVID symptoms have a higher risk of complications associated with metabolic health since significant abnormalities were observed in HbA1c and ferritin levels, in addition to several minor abnormalities in other studied markers in these patients. This might be explained by the temporal proximity to the acute involvement of COVID-19 since even though the patient is still presenting with long COVID symptoms, most of the laboratory markers investigated here showed normalisation in their mean levels in more extended periods of long COVID. This supposed benign evolution can be related to evidence that the number of symptoms tends to decrease over time [18,19,20], although when comparing patients with up to three months and with more than one year of long COVID, we could not observe a significant difference in the number of simultaneous symptoms reported.

It was shown here that BMI measures suggestive of obesity and abnormalities in triglycerides, HbA1c, and ferritin were prevalent in worse clinical long COVID scenarios, such as previous acute hospitalisation and presentation of more symptoms, indicating a worse long COVID involvement, in agreement with other studies [7,21,22]. This prevalence may suggest that patients with long COVID may be more likely to present abnormalities in the markers involved in cardiometabolic health, and consequently, with complications related to MS. It has been hypothesised that acute inflammation driven by SARS-CoV-2 infection could dysregulate metabolic pathways, for instance, through the impairment of insulin signalling, which could lead to abnormalities in cardiometabolic homeostasis [13,23].

Regarding ferritin, it is important to emphasise that the results showed a prevalence of higher levels based on the proximity to the acute phase of COVID-19, as previously presented in the literature [13,24,25]. Thus, the groups of hospitalised patients and those with a shorter period of long COVID had higher ferritin levels than their counter-groups. High ferritin levels have been found to be related to a poor prognosis of acute COVID-19 and may also be elevated in the long COVID-19 phase [24,26]. However, in this study, the levels of ferritin were unexpectedly higher in the group of patients with up to six symptoms of long COVID, indicating an abnormality in this marker in a group with a less severe outcome. This could be explained by the fact that, in this study, the group with fewer symptoms consisted of more patients with a shorter duration of long COVID, leading to a higher prevalence of high ferritin levels in this group, but further investigations are advisable.

The lack of a control group is a limitation of this study. A control group would allow for a comparison between patients with and without prolonged symptoms. Additionally, evaluating screening clinical and laboratory markers may not be enough to detail all the particularities of metabolic health, considering the complexity of long COVID. On the other hand, monitoring the markers involved in metabolism that are widely available certainly enhances our understanding of the metabolic profile of patients with long COVID. To the best of our knowledge, this is the first study to demonstrate a possible metabolic imbalance in patients with up to 632 days of long COVID, highlighting MS-related markers while linking these metabolic abnormalities to the clinical context of long COVID.

Here, interesting clues regarding the relationship between long COVID and metabolic-health-related markers are provided, examining how the metabolic profile is presented in patients with long COVID. Illustrating how common markers in clinical practice, especially those involved in glycaemic and lipid metabolism, relate to the presentation of the disease, by presenting population prevalence patterns, could provide useful insight into possible risk patterns and markers and help to better manage these patients. The findings presented here, together with other evidence [7,11,22], may provide a basis for more robust and detailed investigations in the future.

## 5. Conclusions

This study has examined how the metabolic profile is affected in patients with long COVID, illustrating how common markers in clinical practice relate to the course of the disease. Our main findings indicate that abnormal triglyceride, HbA1c, BMI, and ferritin levels are prevalent in worse long COVID presentations, such as hospitalisation in the acute phase and more concomitant symptoms. This prevalence may suggest a propensity for patients with long COVID to present abnormalities in the markers involved in cardiometabolic health. Therefore, it is recommended that health systems be prepared to receive an increasing number of patients affected by conditions related to MS, given the probable influence of long COVID. It is also suggested that further investigations, especially regarding the cellular metabolic mechanisms shared by MS and long COVID, be conducted in case symptoms persist. Importantly, cohort studies that follow patients with long COVID for an extended period are advisable and could provide a better understanding of how the metabolic profile develops in these patients.

## Figures and Tables

**Table 1:** Clinical and laboratory profile in the study population.

Variable	*n* = 215	Laboratory Metabolic Disturbances ^†^		*p* *
		Yes	No	
Female, n (%)	138 (64.1)	61 (59.8)	77 (68.1)	0.2581
Age, mean ± SD, years	49.6 ± 12.7	51.5 ± 11.6	47.9 ± 13.4	0.0351
18–59 years old, n (%)	161 (74.8)	72 (70.5)	89 (78.7)	0.2216
≥60 years old, n (%)	54 (25.1)	30 (29.4)	24 (21.2)	
Weight, mean ± SD, kg	77.7 ± 16.5	78.3 ± 16	77.1 ± 16.9	0.6089
Height, mean ± SD, m	1.61 ± 0.09	1.62 ± 0.10	1.6 ± 0.08	0.5450
BMI, mean ± SD, kg/m^2^	29.7 ± 5.8	29.9 ± 5.7	29.6 ± 6	0.5952
SBP, mean ± SD, mm/Hg	125.3 ± 14.1	127.3 ± 14.2	123.6 ± 13.8	0.1480
DBP, mean ± SD, mm/Hg	83.9 ± 9.6	84.3 ± 9.8	83.6 ± 9.5	0.6107
Current/Former smoker, n (%)	63 (29.3)	28 (27.4)	35 (30.9)	0.6770
Reported comorbidities, n (%)	77 (35.8)	42 (41.1)	35 (30.9)	0.1569
Hypertension, n (%)	64 (29.7)	36 (35.2)	28 (24.7)	0.1249
Other ^(a)^, n (%)	26 (12)	14 (13.7)	12 (10.6)	0.6255
Hospitalised in acute phase, n (%)	78 (36.2)	43 (42.1)	35 (30.9)	0.1185
Hospitalisation period ^(b)^, mean ± SD, days	19.6 ± 17.1	21.5 ± 19.8	17.4 ± 13.1	0.4101
Hospitalised >7 days ^(b)^, n (%)	64 (82)	37 (86)	27 (77.1)	0.4700
Fatigue ^(c)^, n (%)	184 (85.5)	85 (83.3)	99 (87.6)	0.4857
Dyspnoea ^(c)^, n (%)	178 (82.7)	80 (78.4)	98 (86.7)	0.1533
Muscle weakness ^(c)^, n (%)	168 (78.1)	88 (86.2)	80 (70.7)	0.0100
Muscle pain ^(c)^, n (%)	144 (66.9)	62 (60.7)	82 (72.5)	0.0912
Loss of balance ^(c)^, n (%)	127 (59)	67 (65.6)	60 (53)	0.0826
Chest pain ^(c)^, n (%)	116 (53.9)	52 (50.9)	64 (56.6)	0.4877
Headache ^(c)^, n (%)	116 (53.9)	51 (50)	65 (57.5)	0.3331
Insomnia ^(c)^, n (%)	111 (51.6)	54 (52.9)	57 (50.4)	0.8185
Visual disturbances ^(c)^, n (%)	109 (50.6)	53 (51.9)	56 (49.5)	0.8295
Cough ^(c)^, n (%)	97 (45.1)	42 (41.1)	55 (48.6)	0.3342
Tremor ^(c)^, n (%)	85 (39.5)	45 (44.1)	40 (35.3)	0.2436
Lower limb oedema ^(c)^, n (%)	78 (36.2)	40 (39.2)	38 (33.6)	0.4784
Number of long COVID symptoms, mean ± SD	7 ± 2.7	6.9 ± 2.7	7 ± 2.6	0.9190
≤6 symptoms, n (%)	94 (43.7)	45 (44.1)	49 (43.3)	0.9791
>6 symptoms, n (%)	121 (56.2)	57 (55.8)	64 (56.6)	
Long COVID period, mean ± SD, days	247.7 ± 151.2	241.9 ± 149.5	253 ± 153.2	0.6774
≤90 days, n (%)	35 (16.2)	19 (18.6)	16 (14.1)	0.4832
≤180 days, n (%)	74 (34.4)	38 (37.2)	36 (31.8)	0.4915
>365 days, n (%)	45 (20.9)	21 (20.5)	24 (21.2)	0.9595
LDL-C, mean ± SD, mg/dL	128.7 ± 40	-	-	-
HDL-C, mean ± SD, mg/dL	45.7 ± 9.9	44.8 ± 10.4	46.4 ± 9.5	0.1633
Total cholesterol, mean ± SD, mg/dL	204.3 ± 44.6	-	-	-
Triglycerides, mean ± SD, mg/dL	154.1 ± 101.3	-	-	-
FBG, mean ± SD, mg/dL	99.5 ± 21.1	-	-	-
HbA1c ^(d)^, mean ± SD, %	5.9 ± 0.8	-	-	-
Ferritin, mean ± SD, ng/mL	176.5 ± 159	219.2 ± 189.2	138 ± 113.3	0.0005
ESR, mean ± SD, mm	39.5 ± 25.5	42.3 ± 26.2	36.9 ± 24.7	0.0987
CRP positive, n (%)	27 (12.5)	16 (15.6)	11 (9.7)	0.2674
Total, n (%)	215 (100)	102 (47.4)	113 (52.5)	-

* ANOVA and Mann–Whitney tests were used to compare the normal and non-normal continuous variables, respectively; chi-square and Fisher exact tests were used to compare the categorical variables. Values are expressed as the mean ± standard deviation (SD). ^†^ FBG ≥ 126 mg/dL and/or HbA1c ≥6,5% and/or LDL-c ≥ 160 mg/dL and/or triglycerides ≥ 200 mg/dL: ^(a)^ heart, liver, kidney, or gastric diseases; ^(b)^ n = 78; ^(c)^ shown for ≥four weeks from symptom onset; ^(d)^ n = 150; BMI, body mass index; SBP, systolic blood pressure; DBP, diastolic blood pressure; LDL-C, low-density cholesterol; HDL-C, high-density cholesterol; FBG, fasting blood glucose; HbA1c, glycated haemoglobin A1c; ESR, erythrocyte sedimentation rate; CRP, C-reactive protein.

**Table 2:** Comparison of the metabolic-related variables in the long COVID outcome groups.

Variable	Hospitalised in Acute Phase			Long COVID Period									Number of Long COVID Symptoms		
	Yes	No	*p* *	≤90 Days	>90 Days	*p* *	≤180 Days	>180 Days	*p* *	≤365 Days	>365 Days	*p* *	≤6	>6	*p* *
BMI, mean ± SD, kg/m^2^	30.4 ± 5.3	29.3 ± 6.1	0.0586	28.7 ± 6	29.9 ± 5.8	0.2529	29.2 ± 5.8	30 ± 5.9	0.3977	29.3 ± 5.7	31.5 ± 6	0.0222	28.5 ± 5.8	30.7 ± 5.7	0.0019
≥30 kg/m^2^, n (%)	39 (50)	50 (36.4)	0.0736	15 (42.8)	74 (41.1)	0.9965	32 (43.2)	57 (40.4)	0.8004	64 (37.6)	25 (55.5)	0.0456	24 (25.5)	65 (53.7)	<0.0001
SBP, mean ± SD, mmHg	127.3 ± 16	124.3 ± 12.8	0.2322	127.1 ± 13.1	125 ± 14.3	0.3851	127.2 ± 13.8	124.3 ± 14.2	0.1322	124.9 ± 13.6	127.1 ± 15.9	0.6115	126.7 ± 14.6	124.3 ± 13.7	0.5017
≥130 mmHg, n (%)	38 (48.7)	64 (46.7)	0.8881	17 (48.5)	85 (47.2)	0.9691	41 (55.4)	61 (43.2)	0.1211	79 (46.4)	23 (51.1)	0.6991	45 (47.8)	57 (47.1)	0.9791
DBP, mean ± SD, mmHg	83 ± 9.1	84.4 ± 9.9	0.3173	84.5 ± 7.8	83.8 ± 9.9	0.6832	84.5 ± 8.3	83.6 ± 10.3	0.3926	83.8 ± 9.4	84.2 ± 10.3	0.8287	83.7 ± 9.2	84.1 ± 9.9	0.7570
≥85 mmHg, n (%)	28 (35.8)	52 (37.9)	0.8780	16 (45.7)	64 (35.5)	0.3439	29 (39.1)	51 (36.1)	0.7744	62 (36.4)	18 (40)	0.7932	35 (37.2)	45 (37.1)	0.8921
LDL-C, mean ± SD, mg/dL	126.1 ± 37.7	130.2 ± 41.3	0.8312	141 ± 45.6	126.3 ± 38.5	0.0834	133.6 ± 40.1	126.1 ± 39.8	0.2205	131.3 ± 39.5	118.7 ± 40.6	0.0481	129.6 ± 38.6	128 ± 41.1	0.5626
≥130 mg/dL, n (%)	37 (47.4)	64 (46.7)	0.9678	21 (60)	80 (44.4)	0.1331	39 (52.7)	62 (43.9)	0.2824	65 (38.2)	16 (35.5)	0.8753	49 (52.1)	52 (42.9)	0.2317
HDL-C, mean ± SD, mg/dL	44.2 ± 10.9	46.5 ± 9.2	0.0969	45 ± 12.3	45.8 ± 9.4	0.5368	45.5 ± 11	45.7 ± 9.3	0.7898	46 ± 9.9	44.4 ± 9.9	0.2883	44.7 ± 9.2	46.4 ± 10.4	0.3985
<40 mg/dL, n (%)	28 (35.8)	31 (22.6)	0.0527	14 (40)	45 (25)	0.1068	24 (32.4)	35 (24.8)	0.3043	47 (27.6)	12 (26.6)	0.4572	27 (28.7)	32 (26.4)	0.8281
Total cholesterol, mean ± SD, mg/dL	207.7 ± 45.2	202.4 ± 44.3	0.3135	217.1 ± 49.8	201.9 ± 43.3	0.0619	210.3 ± 45.6	201.2 ± 44	0.1939	206.9 ± 44.9	194.7 ± 42.6	0.0964	203.9 ± 42.9	204.7 ± 46.1	0.9533
≥200 mg/dL, n (%)	42 (53.8)	62 (45.2)	0.2846	20 (57.1)	84 (46.6)	0.3421	39 (52.7)	65 (46)	0.4372	86 (50.5)	18 (40)	0.2730	49 (52.1)	55 (45.4)	0.4045
Triglycerides, mean ± SD, mg/dL	197.7 ± 130.3	129.3 ± 69.5	<0.0001	154.9 ± 94.1	153.9 ± 102.9	0.5899	166.9 ± 125	147.3 ± 86.1	0.3435	153.1 ± 105.4	157.8 ± 85	0.5230	158 ± 113.5	151 ± 91.1	0.9515
≥150 mg/dL, n (%)	41 (52.5)	42 (30.6)	0.0025	14 (40)	69 (38.3)	0.9965	30 (40.5)	53 (37.5)	0.7833	63 (37)	20 (44.4)	0.4637	40 (42.5)	43 (35.5)	0.3644
FGB, mean ± SD, mg/dL	103.3 ± 27.5	97.3 ± 16.1	0.1502	96.2 ± 13.2	100.1 ± 22.3	0.7597	100 ± 20.8	99.2 ± 21.4	0.3669	99.2 ± 20	100.5 ± 25.2	0.6742	98.8 ± 19.7	100 ± 22.2	0.6705
≥100 mg/dL, n (%)	29 (37.1)	44 (32.1)	0.5459	12 (34.2)	61 (33.8)	0.8810	28 (37.8)	45 (31.9)	0.4717	60 (35.2)	13 (28.8)	0.5288	31 (32.9)	42 (34.7)	0.9038
HbA1c, mean ± SD, % ^(a)^	6 ± 0.7	5.8 ± 0.7	0.1528	6.3 ± 0.8	5.8 ± 0.7	0.0080	6 ± 0.9	5.9 ± 0.7	0.3892	6 ± 0.8	5.7 ± 0.7	0.0990	5.7 ± 0.6	6 ± 0.8	0.0421
≥6%, n (%)	31 (49.2)	32 (36.7)	0.1757	19 (76)	44 (35.2)	0.0004	27 (54)	36 (36)	0.0536	52 (48.5)	11 (25.5)	0.0164	25 (38.4)	38 (44.7)	0.5479
Ferritin, mean ± SD, ng/mL	235.3 ± 195.3	143.1 ± 122.8	0.0002	275 ± 236.4	157.4 ± 131.7	0.0082	220.2 ± 205.3	153.6 ± 122.9	0.1145	179 ± 163	167.1 ± 142.7	0.9979	196 ± 172.7	161.4 ± 146.4	0.0370
High ferritin ^(b)^	24 (30.7)	19 (13.8)	0.0051	14 (40)	29 (16.1)	0.0027	21 (28.3)	22 (15.6)	0.0408	36 (21.1)	7 (15.5)	0.5296	19 (20.2)	24 (19.8)	0.9179
ESR, mean ± SD, mm	36.9 ± 22.6	40.9 ± 26.9	0.4429	28.7 ± 6	38.6 ± 25.2	0.2699	42 ± 26.9	38.2 ± 24.7	0.3787	39.2 ± 24.7	40.5 ± 28.4	0.9688	39.8 ± 28.6	39.2 ± 22.9	0.5390
High ESR ^(c)^	58 (74.3)	95 (69.3)	0.5326	27 (77.1)	126 (70)	0.5159	55 (74.3)	98 (69.5)	0.5600	119 (70)	34 (75.5)	0.5847	65 (69.1)	88 (72.7)	0.6725
CRP, positive, n (%)	11 (14.1)	16 (11.6)	0.7629	6 (17.1)	21 (11.6)	0.3710	11 (14.8)	16 (11.3)	0.6011	21 (12.3)	6 (13.3)	0.9390	10 (10.6)	17 (14)	0.5883
Total, n (%)	78 (36.2)	137 (63.7)	-	35 (16.2)	180 (83.7)	-	74 (34.4)	141 (65.5)	-	170 (79)	45 (20.9)	-	94 ()	121 ()	-

* ANOVA and Mann–Whitney tests were used to compare the normal and non-normal continuous variables, respectively; chi-square and Fisher’s exact tests were used to compare the categorical variables. Values are expressed as the mean ± standard deviation (SD): ^(a)^ n = 150; ^(b)^ ferritin ≥160 ng/mL for females <50 years or ≥300 ng/mL for females ≥50 years or ≥300 ng/mL for males; ^(c)^ ESR ≥15 mm for males <50 years or ≥20 for males ≥50 years or ≥20 for females <50 years or ≥30 for females ≥50 years; BMI, body mass index; SBP, systolic blood pressure; DBP, diastolic blood pressure; LDL-C, low-density cholesterol; HDL-C, high-density cholesterol; FBG, fasting blood glucose; HbA1c, glycated haemoglobin A1c; ESR, erythrocyte sedimentation rate; CRP, C-reactive protein.

**Table 3:** Association between the metabolic-related variables and long COVID outcomes.

Risk Variables (n = 150)	Long COVID Outcomes											
	Long COVID Period >90 Days (n = 125)			Long COVID Period >365 Days (n = 43)			Number of Long COVID Symptoms >6 (n = 85)			Fatigue (n = 129)		
	Coefficient	*p*-Value	Odds Ratio	Coefficient	*p*-Value	Odds Ratio	Coefficient	*p*-Value	Odds Ratio	Coefficient	*p*-Value	Odds Ratio
Female gender	−0.4693	0.4882	0.6254	−0.5481	0.2590	0.5781	1.0860	0.0178	2.9624	0.5822	0.3845	1.7900
Age ≥ 60 years	−0.6034	0.3225	0.5469	0.2220	0.6323	1.2486	−0.7495	0.0878	0.4726	−0.9507	0.1056	0.3865
Hospitalisation in acute phase	−1.7704	0.0051	0.1703	−1.3458	0.0035	0.2603	1.2121	0.0082	3.3605	1.2778	0.0805	3.5886
Long COVID period, ≤90 days	-	-	-	-	-	-	−0.9821	0.1029	0.3745	−2.2518	0.0053	0.1052
LDL-C ≥ 130 mg/dL	−0.9950	0.2245	0.3697	−0.3672	0.5795	0.6927	−0.2434	0.6839	0.7839	0.0877	0.9170	1.0916
HDL-C < 40 mg/dL	−1.2671	0.0622	0.2816	−0.2747	0.5727	0.7598	−0.2601	0.5861	0.7709	−1.0827	0.1018	0.3387
Total cholesterol ≥ 200 mg/dL	−0.2851	0.7323	0.7520	−0.3937	0.5515	0.6745	−0.5681	0.3459	0.5666	−0.7048	0.4166	0.4942
Triglycerides ≥ 150 mg/dL	1.2534	0.0833	3.5023	0.6445	0.1554	1.9051	−0.4701	0.2797	0.6249	−1.4015	0.0361	0.2462
FBG ≥ 100 mg/dL	0.3211	0.6416	1.3786	−0.1047	0.8430	0.9006	−0.0473	0.9226	0.9538	1.4034	0.0710	4.0690
HbA1c ≥ 6%	−1.9821	0.0031	0.1378	−1.2536	0.0136	0.2855	0.5892	0.2080	1.8026	0.1412	0.8434	1.1517
High ferritin ^(a)^	−1.6889	0.0134	0.1847	−0.1393	0.8077	0.8699	0.4309	0.4334	1.5386	0.9373	0.2279	2.5531
High ESR ^(b)^	0.2591	0.6855	1.2958	0.4676	0.3248	1.5962	0.3295	0.4478	1.3902	0.2968	0.6313	1.3455
BMI ≥ 30 kg/m^2^	0.3330	0.5840	1.3952	1.0535	0.0169	2.8677	1.1749	0.0040	3.2379	1.1376	0.0865	3.1192
SBP > 130 mmHg	0.5560	0.4241	1.7436	−0.0754	0.8794	0.9273	0.3615	0.4404	1.4354	0.2697	0.6757	1.3096
DBP > 85 mmHg	−0.8934	0.1832	0.4093	0.0351	0.9455	1.0357	−0.1534	0.7488	0.8578	−0.0337	0.9605	0.9668

Probability prediction of long COVID clinical and laboratory outcomes by multiple logistic regression: ^(a)^ ferritin ≥160 ng/mL for females <50 years or ≥300 ng/mL for females ≥50 years or ≥300 ng/mL for males; ^(b)^ ESR ≥15mm for males <50 years or ≥20mm for males ≥50 years or ≥20mm for females <50 years or ≥30mm for females ≥50 years; BMI, body mass index; SBP, systolic blood pressure; DBP, diastolic blood pressure; LDL-C, low-density cholesterol; HDL-C, high-density cholesterol; FBG, fasting blood glucose; HbA1c, glycated haemoglobin A1c; ESR, erythrocyte sedimentation rate.

## Data Availability

The data that support the findings of this study are available on request from the corresponding author, L.F.M.F. The data are not publicly available due to containing information that could compromise the privacy of research participants.

## Supplementary Materials

The following supporting information can be downloaded at: https://www.mdpi.com/article/10.3390/nu15051197/s1, Table S1: Adopted reference values for clinical and laboratory exams.
