# Supplementary material for: Metabolic Profile of Patients with Long COVID: A Cross-Sectional Study
Source: Nutrients. 2023 Feb 27;15(5):1197. doi: 10.3390/nu15051197 (PMC10005061; doi:10.3390/nu15051197)
Supplement: Supplementary file 1 [file nutrients-15-01197-s001.zip › nutrients-2231228-supplementary.pdf]

Supplementary Table S1. Adopted reference values for clinical and laboratory exams.

Exam	Desirable	Increased Risk
BMI, kg/m^2	18,5 – 24,9	≥30
SBP, mm/Hg	<130	≥140
DBP, mm/Hg	<85	≥90
LDL-C, mg/dL	<130	≥160
HDL-C, mg/dL	≥60	<40
Total cholesterol, mg/dL	<200	≥240
Triglycerides, mg/dL	<150	≥200
FBG, mg/dL	65 – 99	≥126
HbA1c, %	4.8 – 5.9	≥6,5
Ferritin, ng/mL	15 – 160[a] / 25 – 300[b]	≥160[a] / ≥300[b]
ESR, mm	<15[c], <20[d], <30[e]	≥15[c], ≥20[d], ≥30[e]
CRP detection	Negative	Positive

(a), females <50 years; (b), males / females ≥50 years; (c), males <50 years; (d), males ≥50 years / females <50 years; (e), females ≥50 years; BMI, body mass index; SBP, systolic blood pressure; DBP, diastolic blood pressure; LDL-C, low-density cholesterol; HDL-C, high-density cholesterol; FBG, fasting blood glucose; HbA1c, glycated haemoglobin A1c; ESR, erythrocyte sedimentation rate; CRP, C-reactive protein.
